# Structure, Mechanical and Thermal Properties of Polyphenylene Sulfide and Polysulfone Impregnated Carbon Fiber Composites

**DOI:** 10.3390/polym11040684

**Published:** 2019-04-15

**Authors:** Dilyus Chukov, Sarvarkhodzha Nematulloev, Mikhail Zadorozhnyy, Victor Tcherdyntsev, Andrey Stepashkin, Dmitry Zherebtsov

**Affiliations:** National University of Science and Technology “MISIS”, 119049, Leninskii prosp, 4 Moscow, Russia; dil_chukov@mail.ru (D.C.); nematulloev.sarvar@yandex.ru (S.N.); priboy38@mail.ru (M.Z.); a.stepashkin@yandex.ru (A.S.); dmitry_zherebtsov@bk.ru (D.Z.)

**Keywords:** carbon fibers, surface modification, polysulfone, polyphenylene sulfide, impregnation

## Abstract

The paper studies new high-temperature thermoplastic impregnated unidirectional carbon fiber composites. The research focuses on the effect of thermal and chemical oxidation of the carbon fibers surface on the interfacial interaction between fibers and polysulfone and polyphenylene sulfide as well as thermal and mechanical properties of the composites. The research reveals the interaction between carbon fibers and the polymer matrix depend both on the type of surface treatment and nature of the polymer. The chemical oxidation of carbon fibers results in good interfacial interaction, and the best mechanical properties were observed for tows impregnated with polyphenylene sulfide.

## 1. Introduction

Thermoplastic polymer-based composites are attractive materials due to their functional properties, such as high thermal stability, crack resistance, impact toughness, and chemical resistance. High efficiency of thermoplastic-based composites in relation to thermoset-based ones is associated with their rapid production, unrestricted shelf life of semi products (prepregs), a possibility of restoring the form of damaged products without any changes in their functional properties, and a high recyclability as the matrix and fibers do not necessarily need to be separated. Carbon-filled composites-based on high-performance engineering thermoplastics, such as polysulfone and polyphenylene sulfide, show promise for many tribological, aircraft and constructional applications [1,2,3,4]. However, these polymers have some drawbacks such as high processing temperature and, especially, low adhesion to carbon fillers [4,5]. It is known that the realization of the mechanical properties of the fibers in the composites mainly depends on the fiber-matrix interfacial interaction [6,7,8]. The most preferable adhesion mechanism is chemical interaction, which ensures the highest strength of the interface. Only in this case, the composite material is a single whole, rather than a mechanical mixture of two incompatible components, as this behavior occurs at adhesive interaction realized exclusively due to mechanical interlocking, or Van der Waals forces. Using carbon fibers (CF) as fillers, it is required to take into account their low adhesion to matrix materials due to chemical inertness of the CF surface. Various methods, such as thermal or chemical modification of carbon fibers surface [9,10,11,12], plasma treatment [13,14], γ-ray irradiation [15], or in situ polymerization of the polymer matrix on the filler surface [16], were applied to improve the interfacial interaction between polymers and carbon fillers.

Previously, we investigated the effect of thermal and chemical oxidation of the carbon fiber surface on the interfacial interaction between CF and ultra-high-molecular-weight polyethylene [17,18]. It was shown that the surface oxidation results in the formation of a continuous polymer film on the fiber surface, providing a significant increase in the mechanical properties of composites [18].

Production of high-temperature thermoplastic-based composites reinforced with carbon fiber has substantially increased over the past few years. In this work, we investigate the structure, thermal and mechanical properties of CF impregnated with high-performance thermoplastic polymers polysulfone (PSU) and polyphenylene sulfide (PPS). Polyphenylene sulfide (PPS) is a thermoplastic polymer consisting of aromatic rings linked by sulfides, a partially crystalline, high-temperature performance polymer. It is polymerized by reacting dichlorobenzene monomers with sodium sulfide at about 250 °C in a high-boiling, polar solvent. Polysulfone is an amorphous thermoplastic polymer in which the main structural chain mostly consists of benzene rings linked together by sulfonyl (―SO_2_―), ether (―O―), and isopropylidene (―C(CH_3_)_2_―) groups. The PSU is prepared by a polycondensation reaction of the sodium salt of an aromatic diphenol and bis(4-chlorophenyl)sulfone. The polymerization is carried out at 130–160 °C under inert conditions in a polar, aprotic solvent, e.g., dimethyl sulfoxide, forming a polyether by elimination of sodium chloride. Due to the aromatic benzene rings in the backbone of the polymers, the PSU and PPS possess an excellent balance of properties, including high-temperature resistance, mechanical properties, chemical resistance, flowability, dimensional stability, and low moisture absorption.

Unidirectional carbon fiber tows impregnated with thermoplastics is a suitable object for evaluating the effect of various methods of CF surface modification on the adhesive interaction between fiber and polymer and on the mechanical properties of the obtained composites [19,20,21]. Taking into account that pre-impregnated carbon fibers are now considered as a promising material for continuous fiber-reinforced thermoplastic-based composites [22], the development and investigation of such composites are of particular interest. The results of our study can broaden knowledge about processing methods, structure, and properties of the PSU- and PPS-based composites.

## 2. Materials and Methods 

**Figure 1:**
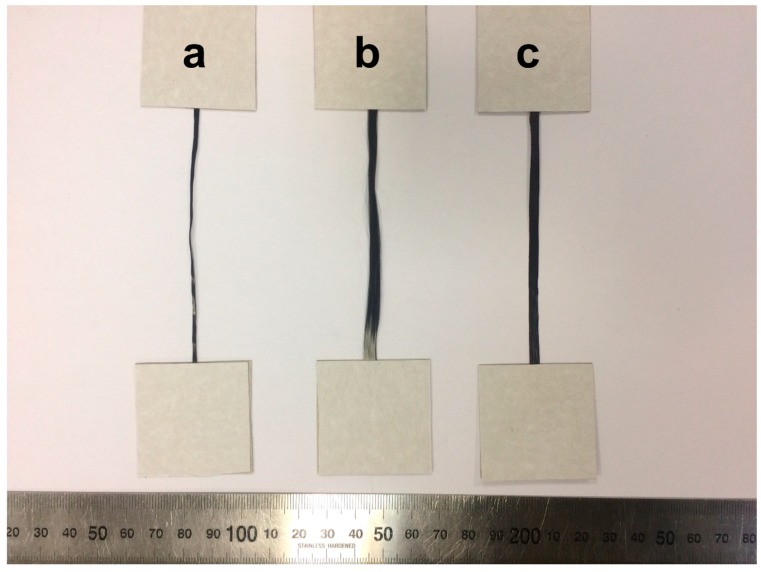
Carbon fibers (**a**) at initial state, (**b**) after thermal treatment, and (**c**) outlook of PPS impregnated fibers.

High strength UKN-5000 (Argon Ltd, Balakovo-1 Russia) carbon fibers (Figure 1a), DIC DSP B-100-C (DIC Corporation, Tokyo, Japan) polyphenylene sulfide, and Ultrason S2010 (BASF SE, Ludwigshafen, Germany) polysulfone were used for the composite formation. Mechanical properties of the raw materials are listed in Table 1.

The conditions for thermal and chemical treatments of the carbon fibers were chosen based on our previous studies [10,12,17]. Chemical oxidation of the fiber surface was performed by direct action of 68.3% nitric acid (HNO_3_) for 3 h at 110 °C. The oxidized fibers were washed with distilled water and dried at 80 °C for 3 h. The thermal oxidation of the carbon fibers was carried out in the air atmosphere at temperatures of 500 °C for 10 min.

It is known that to improve the technological properties of tows, carbon fiber surface is coated with an epoxy-based sizing agent at the last step of fiber production. Sizing for carbon fiber has two prime goals. One goal is to protect the fiber in order to prevent individual filaments from breaking and improve handling of very fine carbon filaments, typically 5 to 7 µm diameter, some of which may stray from the strand. The other goal is to provide compatibility with the molding process, the selection of which can be dictated to a degree by the matrix resin that will be used. The removal of this epoxy-based layer is necessary to achieve strong adhesive interaction between fibers and thermoplastic polymer after impregnation because the epoxy-based layer can play the separation role and prevent the formation of strong bonds at the polymer/fiber interface. As it was shown and discussed in [10,17,18], the chosen surface modification regimes result in the total cleaning of the surface of the fibers from epoxy-based sizing. Figure 1b shows the fibers after oxidation. In particular, the results show that both chemical and thermal treatments cause the same changes in the appearance of the fibers. As it is seen from Figure 1b, the fibers after the treatments look fluffed, individual filaments are partially separated from the tows.

For impregnation, the carbon fibers and polymer powder were held between two heating plates which were heated up to 350 °C. To achieve a homogenous structure of the composites, the external pressure of 5 MPa was applied using a hydraulic press to the plates for more uniform impregnation of the fibers throughout the entire length. The impregnation time was about 3 min, and then the samples were rapidly cooled to room temperature. Figure 1c shows the appearance of the polymer-impregnated fibers. It should be noted that the polymer was deposited on the fiber surface nearly uniformly, no visible nodules were observed. The polymer to fibers ratio in the obtained composites was about 40 wt.% to 60 wt.%.

Structure of the samples was investigated using Hitachi TM-1000 low-vacuum scanning electron microscope (SEM) (Hitachi Ltd, Tokyo, Japan) in a backscattered electron image mode at an accelerating voltage of 15 kV. The uniaxial tensile tests were carried out using Zwick Z020 universal testing machine equipped with 1 and 20 kN load cell and a contact strain measurement system MultiXtens. at a rate of 10 mm/min. The samples span length was 100 mm. To avoid slip/compliance problems prior to the mechanical tests, both ends of the composites were glued to thin cardboard with a size of 60 × 50 mm. The dynamic mechanical properties were characterized with DMA Q800 ((TA Instruments, New Castle, DE, USA) dynamic mechanical analyzer. The specimens for the DMA tests were approximately 3 mm wide, 1 mm thick and 15 mm long. The measurements were performed in a tensile mode at a frequency of 1 Hz, in a temperature range from 30 to 285 °C for the PPS-based composites, and from 30 to 225 °C for the PSU-based composites, the heating rate was of 2 °C/min. Thermal analysis was carried out using NETZSCH DSC 204 F1 differential scanning calorimeter (NETZSCH DSC 204 F1, NETZSCH Group, Selb, Germany) in an argon atmosphere. During the DSC experiments, the PPS-based composites were heated up to 320 °C, at a heating rate of 10 °C/min, then cooled to 35 °C with the same rate, and reheated up to 320 °C. The PSU-based composites were heated up to 210 °C. For all of the tests at least five samples were used.

## 3. Results and discussion

**Figure 2:**
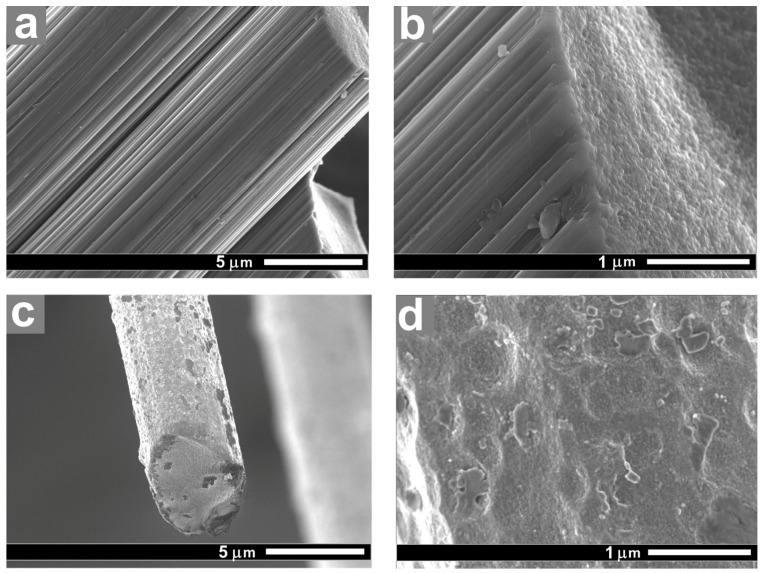
SEM images of the carbon fibers: (**a**,**b**) after nitric acid oxidation (3 h at 110 °C) and (**c**,**d**) after thermal oxidation (10 min at 500 °C).

Structure and morphology of the carbon fibers surface depend significantly on the surface modification conditions [17,23]. SEM studies allow to observe the effect of the modification methods on the carbon fibers structure. Figure 2a,b show the CF surfaces after chemical treatment. SEM images show that after treatment of the fibers in nitric acid, deep etched grooves along the fiber axis were formed. These grooves are the result of removing amorphous carbon and the defective layer from the boundaries of the CF forming fibrils. After thermal treatment of the fibers, the oxidation processes proceed into the depth of the fibers, which leads to the formation of a well-developed surface with craters less than 1 μm in size (Figure 2c,d), the fibers average diameter reduces by 0.3–0.5 mm. In this case, the oxidation of both amorphous and ordered carbon occurs equally. This result indicates that thermal oxidation has a stronger effect on the structure of the fibers than a chemical one, which is manifested in a more pronounced change of the CF surface morphology after thermal treatment.

**Figure 3:**
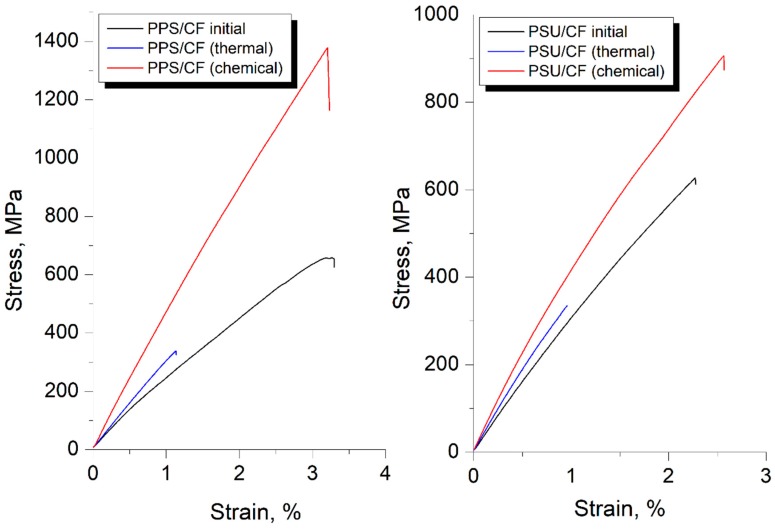
The stress-strain curves for the carbon fiber reinforced PPS- (**left**) and PSU- (**right**) based composites.

The effect of the surface modification methods on the mechanical properties of the composites was studied using tensile tests of the obtained samples. Figure 3 shows typical stress-strain curves for the modified fiber reinforced PPS- and PSU-based composites. The obtained curves are nearly linear with insignificant bending, which appears at a strain of about 0.75 %. Such bending is associated with the deformation unevenness of the individual filament groups. The results of the tensile tests of the initial and modified carbon fibers impregnated with PPS and PSU are given in Table 2. Both the polymer-matrix and the CF surface modification method have a considerable effect on the mechanical properties of the composites. The values of ultimate tensile strength (*σ_uts_*) for the untreated CF impregnated with PPS and PSU (designated as PPS/CF (initial) and PSU/CF (initial), respectively) are nearly equal and are about 649 MPa for PPS/CF (initial) and 629 MPa for PSU/CF (initial) composites.

As it was mentioned previously, thermal treatment provides a stronger effect on the CF surface structure than chemical treatment. Table 2 shows a drastic decrease in tensile strength of the thermally treated CF impregnated both with PPS and PSU (designated as PPS/CF (thermal treatment) and PSU/CF (thermal treatment), respectively), in relation with the untreated CF reinforced ones. The best tensile properties were obtained for the composites reinforced with CF after chemical treatment. In this case, the tensile strength increases up to 1400 MPa for the PPS/CF (chemical treatment) and 920 MPa for the PSU/CF (chemical treatment) composites. It was found that Young’s modulus for the PPS/CF (chemical treatment) composites doubled as compared to that of the untreated CF reinforced ones and reached 52.4 GPa. For PSU/CF (chemical treatment) composites an increase in Young’s modulus (up to 41.6 GPa) is less significant. 

**Figure 4:**
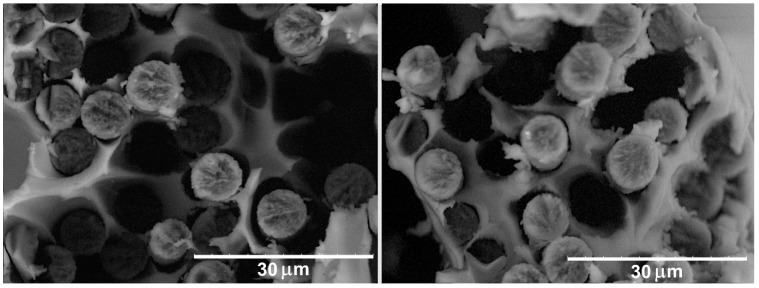
SEM images of the PPS/CF (initial) composites after tensile test.

SEM investigations give an opportunity to explore the interaction between the fibers and polymers and can clarify the observed dependence of the mechanical behavior on the polymer type and the method of the CF surface treatment. Figure 4 shows SEM images of the PPS/CF (initial) composites after the tensile tests. The number of pores formed as a result of the pulling out of the carbon fibers from the polymer matrix shows that there is no strong interfacial interaction between the PPS and the untreated CF. The polymer does not wet the surface of the fibers and due to chemical inactivity of unmodified fibers no strong bonds between the polymer and fiber form. As a result, only the mechanical interaction between the polymer and the CF can be realized in these composites.

**Figure 5:**
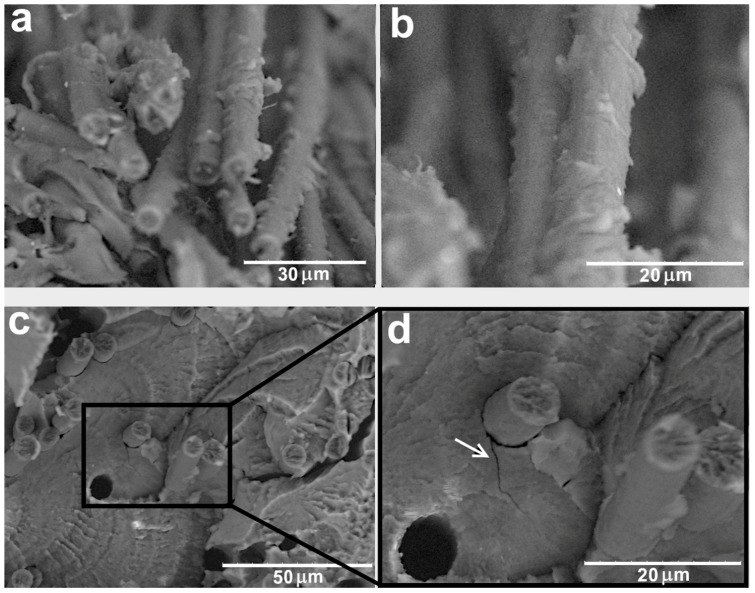
SEM images of the PPS/CF (chemical treatment) composites after tensile test.

It is known that modification of carbon fibers can result in an increase in the concentration of oxygen on their surface, which means an increase in the quantity of oxygen-containing functional groups such as the hydroxyl (-OH), carbonyl (-C=O), and carboxyl (-COOH) groups [24,25]. These functional groups contribute to the formation of the chemical bonds between the CF and the polymer, which results in the formation of a strong interface in the composites. Figure 5a,b show that the chemical treatment of the CF results in the formation of a strong interface with the PPS matrix. Individual filaments are nearly totally coated with the polymer. The destruction of the composites, in this case, does not occur on the fiber-matrix interface but through the polymer matrix breaking. Formation of the strong interface allows a transfer of an applied mechanical load from the polymer matrix to the fibers, which results in a considerable increase in the mechanical properties of the impregnated fibers, see Table 2.

It should be noted that in spite of a good interfacial interaction in the PPS/CF (chemical treatment) composites, SEM shows the existence of some voids formed as a result of the pulling out of the filaments from the polymer matrix (Figure 5c). Moreover, the destruction of the fiber-matrix interfaces can be observed, which is associated with a strong difference in the coefficient of the thermal expansion between CF and PPS. When the material is rapidly cooled, the shrinkage occurs more strongly for the polymer than for the CF, which results in the formation of free space around the filaments. Figure 5d shows that such pores can act as crack nucleation sites (see arrow).

**Figure 6:**
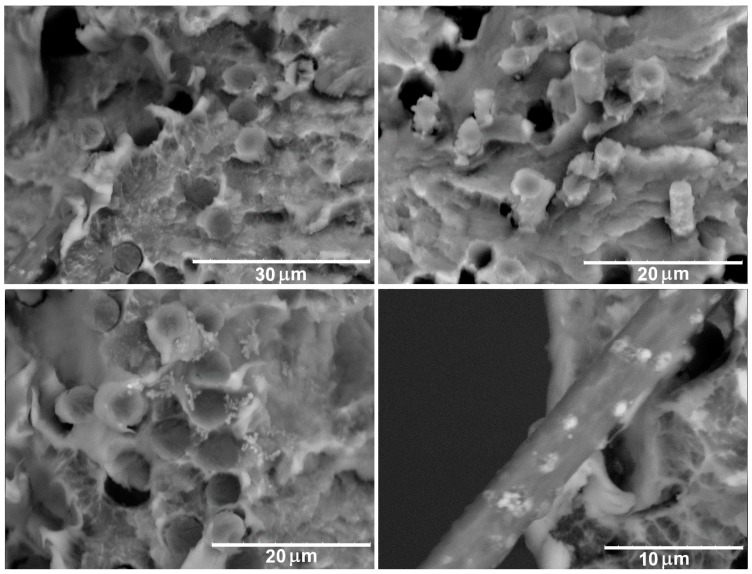
SEM images of the PPS/CF (thermal treatment) composites after tensile test.

The strongest interfacial interaction with the PPS matrix was achieved for the thermally treated CF. As mentioned previously, thermal treatment allows one to significantly affect the CF surface morphology. The thermal treatment results in strong oxidation of the fibers and a large quantity of carbonyl and carboxyl groups forms on the CF surface. These functional groups can provide a strong interaction between the CF and PPS matrix. Figure 6 shows that the polymer completely coats the filaments forming strong fiber-matrix interface. The destruction of the composites, in this case, occurs with an equal probability both through the polymer matrix and the fibers, which is shown by a large quantity of the destroyed ends of the CF located in the same level with the broken surface of the polymer. However, after thermal treatment at 500 °C, a drastic drop in the mechanical properties of the CF occurs [10], which prevents the use of this treatment to form composites with high mechanical properties (see Table 2).

**Figure 7:**
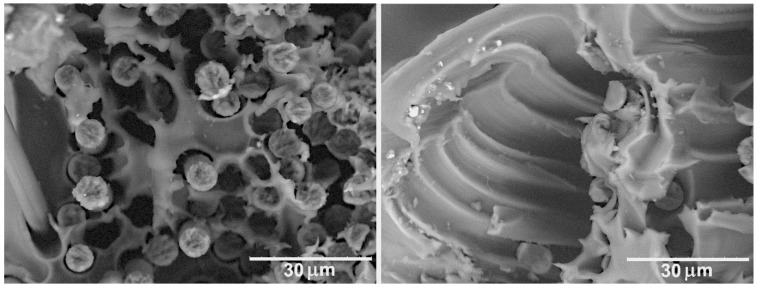
SEM images of the PSU/CF (initial) composites after tensile test.

**Figure 8:**
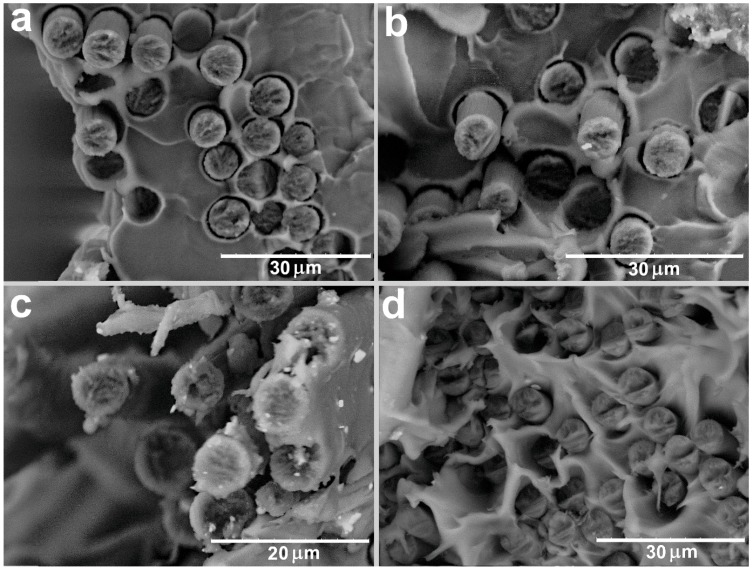
SEM images of (**a**,**b**) the PSU/CF (chemical treatment) and (**c**,**d**) PSU/CF (thermal treatment) composites after tensile test.

For the PSU/CF composites, the observed dependences of the structure on the CF surface modification are similar to those of the PPS/CF composites. The impregnation of the untreated CF with the PSU (Figure 7) does not provide a strong interfacial interaction between the PSU and the untreated CF. Poor interfacial interaction leads to the formation of a number of voids as a result of the pulling out of the fibers from the polymer matrix. Chemical treatment of the CF results in an increase in the adhesion between the CF and PSU (Figure 8a,b). Nevertheless, the adhesion between the chemically treated CF and PSU is lower than that between the chemically treated CF and PPS (see Figure 5). Correspondingly, the mechanical properties of the PSU/CF composites are lower than those of the PPS/CF composites (Table 2).

Thermal treatment of the CF allows forming of a strong interface after the impregnation of CF with PSU (Figure 8c,d). However, as well as for CF impregnated with PPS, a decrease in the mechanical properties of CF after thermal treatment leads to low mechanical properties of such CF after impregnation with PSU.

**Figure 9:**
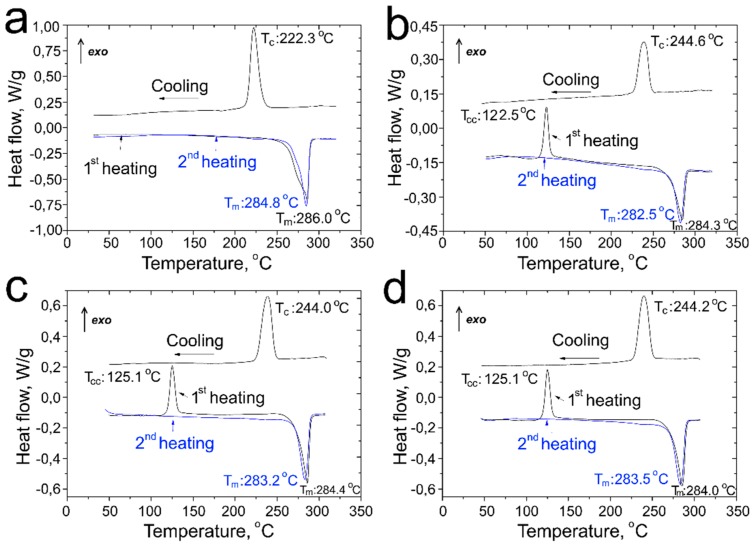
The DSC curves of (**a**) the initial PPS and (**b**) PPS composites reinforced with initial CF, CF after (**c**) chemical and (**d**) thermal treatment.

DSC analyses were performed to study the effect of the CF on the thermal behavior of the PPS- and PSU-based composites. Figure 9 shows the DSC curves of the neat PPS powder and PPS/CF composites. The endothermic peak relates to polymer melting at heating, and the exothermic peak corresponds to the crystallization process during cooling. Table 3 summarizes the melting (*T_m_*), crystallization (*T_c_*), and cold-crystallization (*T_cc_*) temperatures of the experimental samples. *T_cc_* does not exist for the neat PPS, the corresponding thermal effect was observed only in the composite samples as a result of partial amorphization of the polymer due to fast cooling after impregnation. As shown in Figure 9a, the melting temperature of the neat PPS powder is equal to 286.0 °C during the first heating, and it reduces slightly to 284.8 °C during the second heating, correspondingly, the melting temperatures for both first (*T_m1_*) and second (*T_m2_*) heating cycles are given in Table 3. The crystallization temperature of the polymer is 222.3 °C. The difference between the first and second heating cycles is evidence of non-reversible structure transformations in PPS after remelting. The observed difference in the melting temperatures is associated with the difference in the crystallization conditions (temperature and pressure) of the initial PPS powder and the crystallization conditions during DSC measurements. 

The DSC curves of the carbon fiber reinforced composites (Figure 9b) show that the melting temperature of the PPS/CF (initial) composites at the first heating is 284.3 °C, which is 1.7 °C lower than for the initial polymer, and for the second heating the melting temperature is 282.5 °C, which is 2.3 °C lower than for the initial polymer. For the composites reinforced with the modified fibers, the melting point is also lower in comparison with the neat PPS powder. It is known that the melting temperature of a polymer crystal mainly depends on the lamellar thickness and the interaction energy between crystalline and amorphous phases of a polymer. The surface energy of most inorganic crystals has a negligible effect on the melting temperatures because the surface energy is lower in comparison with the volume energy of the crystal. However, the polymer crystals are usually much smaller (several nanometers in size) than most inorganic crystals. That is why the surface energy has a great influence on the full crystal energy [26,27]. As a result, the melting temperature quickly goes down along with the lamellar thickness reduction, so the lamels with a smaller size, or with some defects, melt at lower temperatures than larger and defectless lamels [26]. This dependence is described by the Thomas-Gibbs equation:

(1) $$T_{m}=T_{m}^{0}(1-\frac{2\sigma_{e}}{\Delta H*l})$$

where *T_m_*^0^ is the melting temperature of the lamels of the crystals infinite in sizes, *σ_e_* is the surface energy, *ΔH* is the melting heat, and *l* is the lamellar crystal thickness. 

Presence of differently sized crystallites provides the diffusion character of the polymer melting. High-molecular-weight materials have a wide range of melting temperature in contrast with low molecular ones. Therefore, it can be concluded that the melting temperature reduction of the PPS/CF composites is evidence of the PPS formation lamellas with lower thickness due to the presence of the CF because the CF surface can act as nucleation sides during crystallization. 

The main difference in the DSC curves of the neat PPS and PPS/CF composites is the appearance of an additional exothermic peak at about 125 °C for the composites (Figure 9b–d), which corresponds to cold crystallization of the PPS matrix. As it was reported in [28,29], the PPS exhibits a characteristic phase transition process, namely a rubber–glass transition (α-relaxation). The PPS also experiences a disorder–order transition process (cold crystallization) when heated from room temperature. Partial amorphization of the PPS matrix occurs because of a relatively high cooling rate at the composites production step. Rapid cooling of the PPS composites through its glass transition temperature significantly slows molecular mobility, essentially freezing the structure of the amorphous domains and effectively reducing the molecular alignment in the material. When the composites are heated up to the temperatures higher than PPS glass transition temperature (*T_g_*), the recrystallization process occurs. Provided by rapid cooling non-equilibrium order remains frozen at temperatures lower than *T_g_*_,_ but when heated higher than *T_g,_* crystallizable polymer chains possess sufficient segmental mobility allowing restoring the equilibrium structure of the polymer. 

For the neat PPS, the exothermic peak associated with crystallization at cooling was observed at a temperature of 222.3 °C (Figure 9a). For the PPS/CF composites, this peak shifts towards higher temperatures and is in a range of 244–245 °C (Figure 9b–d), which is 20 °C higher than for the neat polymer. It allows us to propose that the crystallization peak shifting is the result of heterogeneous PPS crystallization on the CF surface. It means that in the presence of the fibers, the PPS crystallization starts at higher temperatures because CF acts as nucleation sites for PPS crystals. Carbon fibers can absorb crystallized material. Therefore, the PPS crystals nucleates primarily on the existed surfaces of foreign crystalline substances because such localizations of the nuclei provide a minimum in free energy [30,31]. The heterogeneous crystallization results in the *T_c_* shifts towards higher temperatures for the PPS/CF composites in relation to neat PPS. 

**Figure 10:**
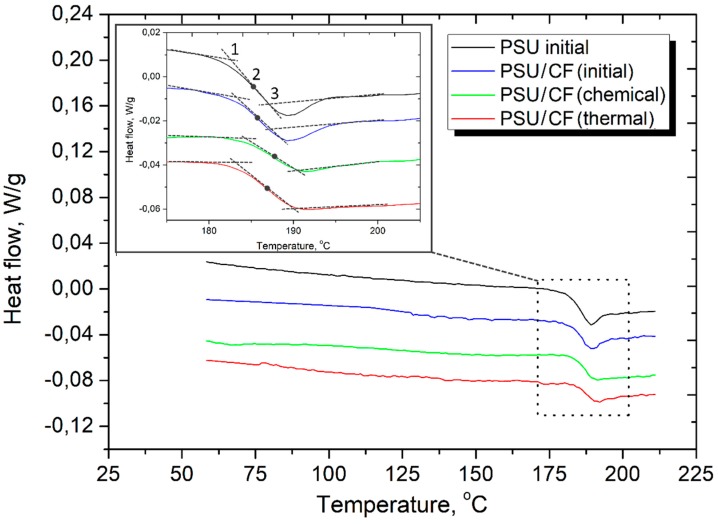
The DSC curves of the neat PSU powder and PSU/CF composites.

The DSC curves of the neat PSU powder and the PSU/CF composites are shown in Figure 10. The drop in the DSC graphs during the increment of temperature represents the glass transition temperature. From the DSC curves, only a single glass transition temperature is observed in a range of 180–190 °C for all the samples. It was found that the onset of the glass transition temperature *T_onset_* for the PSU neat powder is 182.7 °C (Table 4). The carbon fiber reinforcement shifts the polymer glass transition temperature to higher temperatures. The *T_onset_* increases up to 183.2 °C, 183.8 °C, and 184.7 °C for the PSU/CF (initial), PSU/CF (thermal treatment), and PSU/CF (chemical treatment) composites, respectively. The *T_midpoint_* and *T_offset_* of the composites are also higher in comparison with the PSU neat powder. A lower glass transition temperature of the neat PSU generally indicates freer volume in the polymer, which loosens its molecular structure. So, it can be concluded that with the PPS-based composites, carbon fibers can restrict the mobility of the polymer chains. The surface modification improves the fiber–matrix interfacial interaction resulting in more effective immobilization of the polymer chains. Hence, larger *T_g_* of the modified fiber reinforced composites is observed.

**Figure 11:**
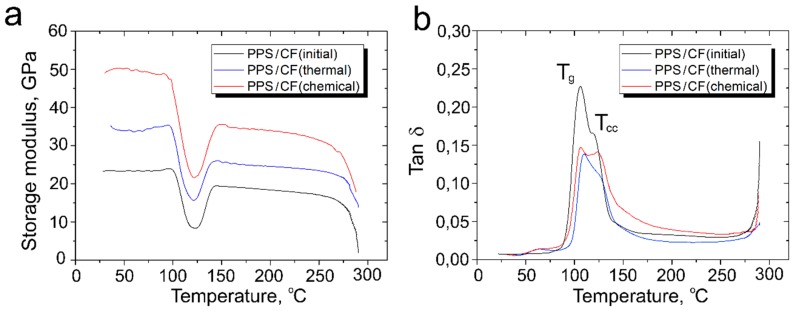
Dependences of (**a**) storage modulus and (**b**) tan δ of the PPS/CF composites on the heating temperature.

The effect of the CF surface modification on the dynamic mechanical response of the polymer-based composites was studied by DMA. Figure 11 shows the storage modulus and tan δ of the PPS-based composites as a function of temperature. Storage modulus indicates a measure of the elastic response of the composite and tan *δ* (*=E″/E′)* is equal to the ratio of the loss modulus (*E″*) to the storage modulus (*E*′), thus representing the damping capacity of a material. 

It is evident that the CF surface modification results in a remarkable increase in the storage modulus of the PPS-based composites. Thus, *E′* of the PPS/CF (initial) composites is 23.4 GPa at 25 °C, and it increases up to 38.5 GPa and 50.2 GPa for the PPS/CF (thermal treatment) and PPS/CF (chemical treatment) composites, respectively. At a temperature range of 100–120 °C, the storage modulus was drastically decreased for all the composites. The *E′* decreases by roughly 57–65% for all the composites, but the modified fiber reinforced composites. The decrease is less than for the initial fiber reinforced composites. The sharp decrease in the storage modulus corresponds to the α-relaxation of the amorphous regions in the PPS, and it is attributed to an energy dissipation phenomenon involving cooperative motions of the polymer chains. The storage modulus starts to increase again at temperatures around 125 °C, which is the result of the cold crystallization of the PPS, which was also observed in the DSC curves. A further increase in the temperature up to the polymer melting point results in a slight decrease in the *E*′ values.

It is well known that mechanical behavior is influenced by the degree of crystallinity. Indeed, the crystalline phase, where the macromolecules are closely packed to each other, strengthens the amorphous phase [32,33]. As a consequence, a polymer with a higher crystalline degree has a higher elastic modulus in the glassy state. Beyond *T_g_*, the difference between a storage module of an amorphous polymer and a crystallized one can reach one to two decades [34]. The structural order in the crystallized material hinders the relaxation of its macromolecular chains. As a consequence, the storage modulus slightly decreases with *T_g_* up to a plateau in the rubbery state. The effect of the temperature on the modulus is even more pronounced for the amorphous polymer. The storage modulus of the amorphous polymer decreases strongly in a temperature range around *T_g_*. Just above the *T_g_*, the modulus increases due to the onset of cold-crystallization of the polymer. Cold crystallization occurs when the polymer chains are quenched (quickly cooled) into a highly disordered state. On heating above the *T_g,_* these chains gain sufficient mobility to rearrange into crystallites that causes a sharp increase in the modulus magnitude. The described behavior of the storage modulus around *T_g_* was reported for various semi-crystalline polymers and polymer-based composites [28,29,34].

The ratio of the loss to the storage modulus (tan *δ*) vs. the temperature is shown in Figure 11b. An abrupt increase of tan *δ* was found for the PPS/CF composites with a rapid decrease of the storage modulus. For all the composites, a double peak of the tan δ in a temperature range of 100–130 °C was observed. The most intense peak (α-relaxation, *T_g_*) corresponds to an amorphous chain segment motion, whereas the second peak corresponds to a crystalline chain segment motion (cold crystallization, *T_cc_*) of the PPS. The CF reinforcement decreases the free volume and inhibits the mobility of the matrix chains. With increasing fiber-matrix interfacial interaction, the restrictions on mobility become stronger leading to higher *T_g_* values. The surface modification improves the fiber–matrix interfacial interaction leading to a more effective immobilization of the polymer chains. Hence, *T_g_* of the modified fiber reinforced composites increased. Thus, *T_g_* of the PPS/CF (initial) composites is 105.4 °C, and it increases up to 106.5 °C and 109.2 °C for the PPS/CF (thermal treatment) and the PPS/CF (chemical treatment) composites, respectively. Cold crystallization temperature *T_cc_* also increases by 3–3.5 °C for the modified fiber reinforced PPS composites. These results are consistent with the DSC results.

A larger area under the α-relaxation peak in the tan *δ* curves of the PPS/CF (initial) composites indicates that the molecular chains exhibit a higher degree of mobility and thus better damping properties. The area under this peak for the modified fiber reinforced composites seems to be slightly smaller. Another interesting feature is the broadening and diminution of the height of tan *δ* of the PPS/modified fibers composites, the effects that were previously reported for some nanofiller-reinforced PPS composites [35,36]. These phenomena are found to be more pronounced in case of oxidized fibers, which should be related to a stronger fiber-matrix interaction.

**Figure 12:**
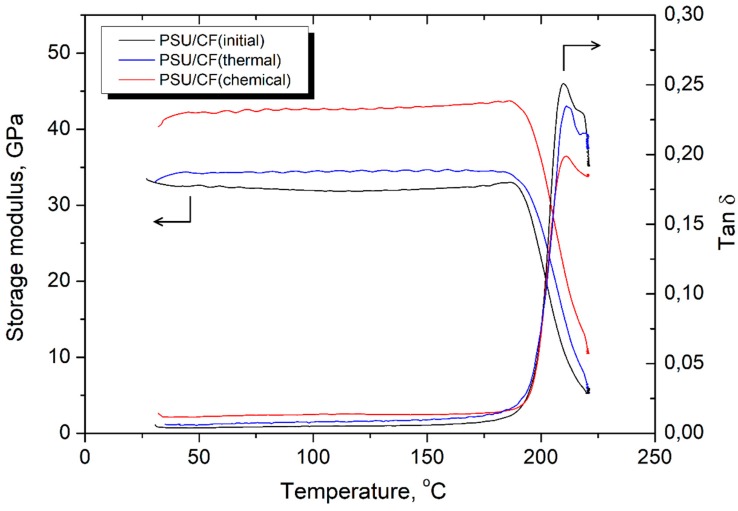
The storage modulus and tan δ of the PSU/CF composites as a function of temperature.

Figure 12 shows the storage modulus and tan δ of the PSU-based composites as a function of temperature. It was found that the CF surface modification results in a remarkable improvement in the storage modulus of the PSU-based composites. The *E′* of the PSU/CF (initial) composites is 32.5 GPa at 25 °C, and it increases up to 34.2 GPa and 42.3 GPa for the PSU/CF (thermal treatment) and the PSU/CF (chemical treatment) composites, respectively. The plateau of the storage modulus for all the composites can be observed up to a glass transition temperature of the PSU where a sharp decrease in the storage modulus occurs. For the PSU/CF (initial) composites, a larger area under the α-relaxation peak in the tan *δ* curves was observed. The area under this peak for the modified fiber reinforced composites is smaller, which also should be related to a stronger fiber-matrix interaction [37,38,39].

## 4. Conclusions

The effect of thermal and chemical surface modifications of carbon fibers on the structure and properties of fibers after impregnation with polyphenylene sulfide and polysulfone was studied. Both chemical and thermal oxidation of carbon fibers allows improvement of interfacial interaction between the fibers and the polymer matrix. The improved interfacial interaction results in a considerable increase in the mechanical properties of the modified carbon fibers reinforced composites in relation to the composites containing untreated fibers. The best mechanical properties were obtained for the PPS-based composites reinforced with carbon fibers after chemical modification in nitric acid (HNO_3_) for 3 h. The ultimate strength and Young’s modulus of these materials double in comparison with those for the polymers reinforced with the untreated fiber. Thermal treatment provides better adhesion of the fiber-matrix interaction. However, the mechanical properties of carbon fibers after thermal treatment decrease drastically, which prevents the use of this treatment to form impregnated CF with high mechanical properties.

It was found that a relatively high cooling rate at the composites production step results in partial amorphization of the PPS matrix. When the composites are heated up to the temperatures higher than the PPS glass transition temperature (*T_g_*), the recrystallization process occurs. The storage modulus magnitude sharply decreased at heating due to α-relaxation of the amorphous regions in the PPS. The storage modulus starts to increase again at temperatures around 125 °C, which is the result of cold crystallization of the PPS matrix. For the PSU-based composites, the plateau of the storage modulus for all the composites was observed up to a glass transition temperature of the PSU. The surface modification improves the fiber–matrix interfacial interaction, resulting in more effective immobilization of the polymer chains. Hence, a larger *T_g_* of the modified fiber reinforced composites was observed. For both PPS/CF and PSU/CF composites CF surface modification results in a remarkable improvement in the thermal and mechanical properties in comparison with the untreated fiber reinforced composites.

## Figures and Tables

**Table 1:** Properties of the initial materials.

	*σ_uts_*, MPa	*E*, GPa	*σ_0,2_*, MPa	*d*, μm
Polyphenylene sulfide	110	3.7	100	20
Polysulfone	75	2.6	50	300
UKN-5000 carbon fibers	4000	250	-	7.5

***σ_t_***: ultimate tensile strength, ***E***: Young’s modulus, ***σ_0,2_***: yield strength, ***d***: average particle size (for polymers) or average filaments diameter (for fibers).

**Table 2:** Mechanical properties of the polymer impregnated carbon fibers composites.

	*σ_uts_*, MPa	*E*, GPa	*ε*, %
PPS/ CF (initial)	649 ± 47.5	25.4 ± 1.9	3.4 ± 0.6
PPS/CF (thermal treatment)	329 ± 30.3	37.8 ± 2	1.5 ± 0.4
PPS/CF (chemical treatment)	1400 ± 82.3	52.4 ± 4.8	3.3 ± 0.4
PSU/CF (initial)	629 ± 21.1	33.1 ± 2.5	2.3 ± 0.2
PSU/CF (thermal treatment)	346 ± 7	36.3 ± 1.8	1.7 ± 0.3
PSU/CF (chemical treatment)	920 ± 54.5	41.6 ± 2.1	2.4 ± 0.4

***σ_uts_***: ultimate tensile strength, ***E***: Young’s modulus, ***ε***: elongation at break, CF: carbon fibers.

**Table 3:** Results of DSC analysis of the PPS/CF composites.

	*T_m1_*	*T_m2_*	*T_cc_*	*T_c_*
PPS neat powder	286.0 ± 0.1	284.8 ± 0.2	-	222.3 ± 0.3
PPS/ CF (initial)	284.3 ± 0.1	282.5 ± 0.1	122.5 ± 0.2	244.6 ± 0.2
PPS/ CF (thermal treatment)	284.4 ± 0.2	283.2 ± 0.2	125.1 ± 0.3	244.0 ± 0.3
PPS/CF (chemical treatment)	284.0 ± 0.1	283.5 ± 0.2	125.1 ± 0.2	244.2 ± 0.2

*T_m1_*—melting point during 1st heating,_-_*T_m2_*—melting point during 2nd heating, *T_cc_*—cold-crystallization temperature, *T_c_*—crystallization temperature.

**Table 4:** Glass transition temperature of the PSU/CF composites.

	Glass Transition Temperature, °C		
	*T_onset_*	*T_midpoint_*	*T_offset_*
PSU neat powder	182.7 ± 0.2	185.3 ± 0.1	187.1 ± 0.2
PSU/CF (initial)	183.2 ± 0.1	186.2 ± 0.2	188.2 ± 0.3
PSU/CF (thermal treatment)	183.8 ± 0.3	186.9 ± 0.2	189.9 ± 0.2
PSU/CF (chemical treatment)	184.7 ± 0.2	187.2 ± 0.1	190.5 ± 0.2
